# Ventricular dispersion and repolarization in Marfan syndrome: A clinical analysis

**DOI:** 10.1016/j.hroo.2025.06.025

**Published:** 2025-07-05

**Authors:** Raphael Spittler, Tim Salzbrunn, Andreas Metzner, Andreas Rillig, Yskert von Kodolitsch, Ruben Schleberger, Julia Moser, Konstantin Krieger, Boris A. Hoffmann

**Affiliations:** 1 Center of Cardiology, Department of Cardiology II – Electrophysiology, University Medical Center Mainz, Mainz, Germany; 2 Department of Cardiac Electrophysiology, University Heart and Vascular Center, University Hospital Hamburg-Eppendorf, Hamburg, Germany; 3 Department of Cardiology, University Heart and Vascular Center, University Hospital Hamburg-Eppendorf, Hamburg, Germany; 4 Asklepios Medical Center Harburg, Hamburg, Germany

**Keywords:** Marfan syndrome, T-peak–T-end, Ventricular dispersion, Arrhythmia, Mortality, Repolarization

## Abstract

**Background:**

Patients with Marfan syndrome (MFS) are at an increased risk of ventricular arrythmia (VA) and death.

**Objective:**

This retrospective observational study aimed to assess the role of electrocardiographic parameters of repolarization and dispersion in risk stratification.

**Methods:**

Baseline 12-lead electrocardiograms were obtained from consecutive patients with MFS treated at a tertiary care specialized outpatient clinic and from age- and sex-matched controls. In patients with MFS, we studied the association of established parameters of repolarization and dispersion with a combined primary end point of VA or all-cause mortality using Cox regression analysis.

**Results:**

A total of 89 patients with MFS (mean age 42 ± 14 years; 54 (61%) women) and 92 controls were included in the analysis. The mean QRS duration (97 ms vs 87 ms) was longer, and the mean corrected QT interval (411 ms vs 379 ms) and T-peak–T-end interval (67 ms vs 62 ms) were also longer in patients with MFS. During a median follow-up of 5.05 years (interquartile range 3.43–10.0 years), 11 patients with MFS (12%) had VA or died. In univariable analyses, male sex (hazard ratio [HR] 5.01; 95% confidence interval [CI] 1.051–23.87), history of atrial fibrillation (HR 9.51; 95% CI 2.51–36.01), larger left atrial volume (HR 1.35 per 10-mL increase; 95% CI 1.12–1.64 per 10-mL increase), and higher N-terminal pro–B-type brain natriuretic peptide levels (HR 1.05 per 100-pg/mL increase; 95% CI 1.03–1.07 per 100-pg/mL increase) were associated with the primary end point, but QRS duration, corrected QT interval, and T-peak–T-end interval or their derivatives were not associated with the primary end point.

**Conclusion:**

Electrocardiographic parameters of repolarization and dispersion are altered in patients with MFS, but their role in risk stratification may be limited.

**Key Findings**

▪ Patients with Marfan syndrome (MFS) are at high risk for ventricular arrhythmias and sudden cardiac death.
▪ Electrocardiographic parameters of repolarization and dispersion are altered in patients with MFS, but their role in risk stratification may be limited.
▪ Patients with MFS are at an increased risk of arrhythmogenic syncope, and those with MFS and syncope are at an increased risk of sudden cardiac death.
▪ N-terminal pro–B-type brain natriuretic peptide levels were associated with a combined primary end point of ventricular arrhythmias or death in multivariable Cox regression analysis, whereas left atrial volume, male sex, history of atrial fibrillation, and normalized T-peak–T-end dispersion were associated only in univariable Cox regression analysis.

## Introduction

Marfan syndrome (MFS) is a genetic disorder that affects connective tissue and leads to a wide range of clinical manifestations affecting the skeletal, ocular, cardiovascular, and pulmonary systems. In addition to the typical cardiovascular risks associated with MFS, such as aortic dissection, there is an increased propensity for arrhythmias, including atrial fibrillation, ventricular tachycardia (VT), and premature ventricular contractions. Such arrhythmias are not uncommon in patients with MFS and are clinically significant because they increase the risk of sudden cardiac death (SCD), contributing to higher rates of morbidity and mortality.^1^, ^2^, ^3^

Previous studies have established various electrocardiographic (ECG) parameters as markers of transmural, apicobasal, or global dispersion of repolarization.^4^^,^^5^ Spatial dispersion has been found to be associated with life-threatening ventricular arrhythmias. Studies have also found an association between ECG parameters and arrhythmogenicity as well as SCD in populations with hypertrophic cardiomyopathies, Brugada syndrome, and reduced left ventricular ejection fraction (LVEF).^6^, ^7^, ^8^ Gupta et al^6^ proposed the ratio of Tpeak-Tend (TpTe) to QT as an arrhythmogenic index associated with a high risk of SCD. This normalization of the TpTe interval in relation to the QT interval makes TpTe comparable despite dynamic physiological changes in heart rate or other conditions that may influence the QT interval.^6^^,^^9^

The objective of this study was to evaluate depolarization, repolarization, and dispersion of ventricular repolarization in a cohort of patients with MFS.

## Methods

### Study population

This is a retrospective, observational, single-center study that included patients who were screened and examined at our specialized outpatient clinic for MFS at the University Heart and Vascular Center Hamburg between December 2013 and September 2017. The diagnosis of MFS was based on the revised Ghent criteria and genetic testing for the *FBN1* (*fibrillin-1*) mutation.^10^ The control group comprised patients treated in the chest pain unit of the University Heart and Vascular Center Hamburg for nonspecific chest complaints between January and August 2018. Because of the difficulty in assessing ECG parameters of ventricular dispersion in the presence of atrial fibrillation or left bundle branch block, these patients were excluded. Furthermore, patients with a follow-up time of less than 7 days were excluded.

The study was approved by the local institutional review board and conducted in accordance with the Declaration of Helsinki on human research. Informed consent was obtained from all participants. The study was conducted and reported in accordance with the STROBE (Strengthening the Reporting of Observational Studies in Epidemiology) guidelines.

### Baseline characteristics and echocardiography

Baseline clinical characteristics were assessed in our MFS outpatient clinic and in the control group at the time of presentation to the chest pain unit. The day of the first documented ECG in patients with MFS and the first presentation in our chest pain unit were each defined as day 0 for follow-up. Clinical examination and patient interview were conducted, focusing on direct and indirect evidence of rhythm events. Transthoracic echocardiography, 12-lead resting ECG, and 24-hour Holter ECGs were performed, and blood samples were collected for further analysis. LVEF, aortic root diameter, and chamber quantification were measured using transthoracic echocardiography in the 2- and 4-chamber views (iE33, Philips Medical Systems, Eindhoven, The Netherlands).

### Assessment of ECG parameters and evaluation of 4 distinct parameters of repolarization and repolarization dispersion

**Figure 1:**
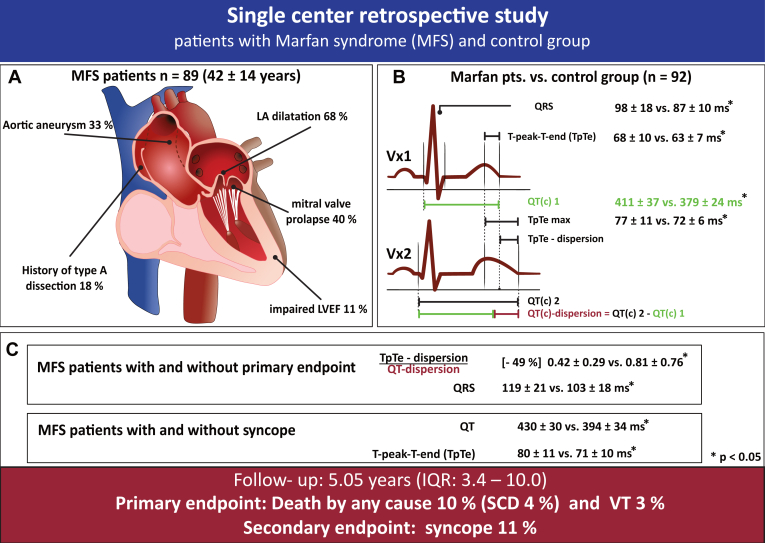
**A:** Distribution of morphological changes in the heart and adjacent structures among patients with Marfan syndrome (MFS). **B:** Overview of the methods used to calculate repolarization and depolarization parameters, along with comparative results between patients with MFS and the control group. **C:** Summary of overall clinical end points and the association between different depolarization markers and both the primary and secondary end points. IQR = interquartile range; LA = left atrial; LVEF = left ventricular ejection fraction; pts. = patients; QTc = corrected QT; SCD = sudden cardiac death; VT = ventricular tachycardia.

Resting 12-lead ECGs were recorded at a paper speed of 25 mm/s and an amplification of 10 mm/mV (CS-200, Schiller Inc., Baar, Switzerland). ECG intervals were measured manually using the technical drawing program Datinf Measure (Datinf GmbH, Tübingen, Germany). The tangent method was used to define the end of the T wave and estimate the QT interval.^11^ We used either the Bazett formula (heart rate between 60–100 bpm) or the Fredericia formula (heart rate <60 or >100 bpm) to correct the QT interval (QTc interval).^12^ We evaluated the following 4 ECG parameters of repolarization dispersion: TpTe, TpTe dispersion (TpTeD), TpTe maximum, and the ratio of Tpeak-Tend-dispersion to QT(c)-dispersion (TpTeD/QT(c)D) (Figure 1).

TpTe was measured from the peak of the T wave to the end of the T wave. The apicobasal TpTeD was calculated as the difference between the longest and shortest TpTe intervals across leads V_1_–V_6_. QTc dispersion (QTcD) was measured as the difference between the longest and shortest QTc intervals across leads V_1_–V_6_. TpTe maximum was defined as the interval between the first T-wave peak and the last T-wave end across leads V_1_–V_6_. This parameter captures the full range of the TpTe interval across all leads (Figure 1).

To account for changes in heart rate and enable a more accurate comparison of TpTe changes independent of QT time, heart rate, and beat-to-beat variability, we calculated the TpTe/QT and TpTe/QTc ratios, as well as the TpTeD/QT(c)D ratio.^6^ Dispersion is typically measured as the interlead variability of the QT or TpTe intervals.^13^

### Follow-up and clinical end points

The primary end point of the study was all-cause mortality and VT. In addition, we assessed syncope events, which may indicate arrhythmogenic events. All patients diagnosed with MFS attended regular follow-up appointments at our specialized outpatient clinic as clinically indicated. We also conducted structured follow-up telephone interviews. We systematically documented information on patient deaths, including SCD occurring outside the hospital as well as during the hospital stay. Notification of these events was obtained from relatives, general practitioners, or hospital records. The classification of the end point was based on clinical data; no postmortem autopsies were performed.

The aim of the study was to identify asymptomatic rhythm events through 24-hour Holter ECG monitoring at each follow-up visit. Additional follow-up visits were scheduled for patients who presented with symptoms suggestive of arrhythmias.

For this study, patients in the control group underwent systematic assessments either at the outpatient clinic or through structured telephone interviews. Further ECG or Holter monitoring was initiated only if new symptoms or cardiac diagnoses emerged.

### Statistical analysis

Baseline characteristics were summarized descriptively for patients with MFS and the control group. Continuous variables are presented as mean ± standard deviation. Between-group differences in continuous variables were tested using the Student *t* test or Mann-Whitney *U* test, as appropriate. A test for normal distribution was performed using the Shapiro-Wilk test. Categorical variables are presented with absolute numbers and relative frequency. Between-group differences in categorical variables were analyzed using the Fisher exact test. Categorical variables were compared using hazard ratios with 95% confidence intervals. Variance was tested using the F test. Correlations were calculated using the Spearman rank correlation coefficient. All significance tests were 2-tailed, with the null hypothesis rejected at *P* < .05.

Receiver operating characteristic (ROC) analysis with area under the curve (AUC) was performed to determine the sensitivity and specificity of parameters associated with the end points. Univariable predictors were assessed using hazard ratios calculated with the Cox regression hazards model. A multivariable Cox regression analysis was performed, including covariables that were significant in the univariable analysis. A post hoc power analysis was performed to determine whether the sample size was sufficient to detect the measured effects.

Statistical analysis was performed using the R programming language (R Foundation for Statistical Computing, R Development Core Team, Vienna, Austria).

## Results

### Study population

Initially, 134 patients with suspected MFS were referred to our specialized outpatient clinic. Using the revised Ghent criteria, the diagnosis of MFS was confirmed in 105 patients. Of those, 16 patients were excluded from the analysis because of follow-up of less than 7 days.

Therefore, the study population consisted of 89 patients with a verified diagnosis of MFS (mean age 42 ± 14 years; 54 (61%) female) (Table 1). All 76 genetically tested patients carried a pathogenic variant of *FBN1*. The control group consisted of 92 almost healthy individuals (mean age 40 ± 7 years; 48 (52%) women). The results are presented in the following order: First, we evaluated ECG alterations in patients with MFS compared to the control group. Second, we compared clinical and ECG parameters in patients with and without the combined end point of all-cause mortality or VT. Third, we conducted an exploratory evaluation of patients with MFS and syncope.

**Table 1:** Baseline characteristics

Parameter	Patients with MFS (n = 89)	Control group (n = 92)	*P*
Age (y)	42 ± 14	40 ± 7	.8
Female sex	54 (61)	48 (52)	.3
Height (m)	1.85 ± 0.11	1.74 ± 0.1	<.001
Weight (kg)	81 ± 22	76 ± 16	.2
Body mass index (kg/m^2^)	23 ± 5	25 ± 5	.002
LVEDD (mm)	54 ± 9	47 ± 4	<.001
LA volume (mL)	55 ± 27	41 ± 9	<.001
Coronary heart disease	6 (7)	1 (1)	.06
Hypertension	13 (15)	10 (11)	.5
Diabetes	5 (6)	1 (1)	.1
Hyperlipidemia	3 (3)	10 (11)	.08
History of type A aortic dissection	16 (18)	0 (0)	–
LVEF			
Normal	79 (89)	79 (85)	.6
Mildly reduced	2 (2)	2 (2)	.9
Moderately reduced	5 (6)	0 (0)	–
Severely reduced	2 (2)	0 (0)	–
Mitral valve prolapse	36 (40)	0 (0)	–
Aortic aneurysm	29 (33)	0 (0)	–
History of atrial fibrillation	15 (14)	0 (0)	–
Aortic valve conduit	11 (12)	0 (0)	
NT-proBNP level (pg/mL)	532 ± 1276	59 ± 56	.001
TSH level (μU/mL)	2.09 ± 1.85	1.63 ± 0.98	.04
LDL cholesterol level (mg/dL)	101 ± 33	114 ± 35	.05
Aortic diameter index	1.86 ± 0.42	–	–

Values are presented as mean ± standard deviation or n (%).

LA = left atrial; LDL = low-density lipoprotein; LVEDD = left ventricular end-diastolic diameter; LVEF= left ventricular ejection fraction; MFS = Marfan syndrome; NT-proBNP = N-terminal pro–B-type brain natriuretic peptide; TSH = thyroid-stimulating hormone.

### ECG in patients with MFS vs control group

Patients with MFS had a longer QRS duration (97 ± 18 ms vs 87 ± 10 ms; *P* < .001) and longer QT (385 ± 38 ms vs 353 ± 29 ms; *P* < .001) and QTc (411 ± 37 ms vs 379 ± 24 ms; *P* < .001) intervals, both measured in lead V_5_, than did control patients (Table 2). TpTe intervals were also longer in patients with MFS across all precordial leads (lead V_5_: 67 ± 10 ms vs 62 ± 7 ms; *P* <.001). TpTe maximum was longer in patients with MFS (77 ± 11 ms vs 72 ± 6 ms; *P* = .001). However, TpTe normalized to QT time was comparable between the 2 groups. There was no significant difference in QTcD (35 ± 25 ms vs 30 ± 20 ms; *P* = .3) and TpTeD (*P* = .6) between the 2 groups (Table 2).

**Table 2:** Electrocardiographic parameters of repolarization and dispersion

Parameter	Patients with MFS (n = 89)	Control group (n = 92)	*P* (MFS vs control)	Patients with MFS and an end point (death + VT) (n = 11)	Patients with MFS and syncope (n = 10)
QRS duration (ms)	98 ± 18	87 ± 10	<.001	119 ± 22	109 ± 26
QT interval (ms)	397 ± 35	375 ± 28	<.001	415 ± 30	430 ± 30
QTc interval (ms)	424 ± 27	401 ± 23	<.001	433 ± 30	435 ± 18
QT dispersion (ms)	32 ± 21	28 ± 18	.2	41 ± 17	36 ± 19
QTc dispersion (ms)	35 ± 24	31 ± 19	.2	43 ± 20	37 ± 19
TpTe V_1_ (ms)	68 ± 10	60 ± 6	<.001	66 ± 7	70 ± 6
TpTe V_2_ (ms)	71 ± 11	67 ± 8	.03	69 ± 12	70 ± 14
TpTe V_3_ (ms)	72 ± 11	68 ± 7	.01	74 ± 12	80 ± 11
TpTe V_4_ (ms)	70 ± 11	65 ± 7	.004	70 ± 9	75± 15
TpTe V_5_ (ms)	68 ± 10	63 ± 7	< .001	67 ± 11	73 ± 14
TpTe V_6_ (ms)	66 ± 9	61 ± 6	< .001	67 ± 9	71 ± 9
TpTe dispersion (ms)	16 ± 7	15 ± 6	.6	14 ± 8	17 ± 8
TpTe maximum (ms)	77 ± 11	71 ± 6	< .001	76 ± 11	74 ± 12
TpTe/QT V_1_	0.18 ± 0.03	0.18 ± 0.03	.4633	0.17 ± 0.03	0.18 ± 0.01
TpTe/QT V_2_	0.19 ± 0.03	0.2 ± 0.03	.089	0.17 ± 0.03	0.16 ± 0.03
TpTe/QT V_3_	0.19 ± 0.03	0.19 ± 0.03	.338	0.19 ± 0.03	0.19 ± 0.02
TpTe/QT V_4_	0.18 ± 0.03	0.19 ± 0.02	.4388	0.17 ± 0.03	0.17 ± 0.03
TpTe/QT V_5_	0.18 ± 0.03	0.18 ± 0.02	.6711	0.17 ± 0.02	0.17 ± 0.03
TpTe/QT V_6_	0.17 ± 0.02	0.17 ± 0.02	.8818	0.16 ± 0.02	0.17 ± 0.02
TpTe-dispersion/QT-dispersion	0.76 ± 0.73	0.71 ± 0.46	.4896	0.42 ± 0.29	0.82 ± 0.92
TpTe V_1_/QTc (ms)	0.17 ± 0.03	0.17 ± 0.02	.5	0.16 ± 0.02	0.17 ± 0.02
TpTe V_2_/QTc (ms)	0.18 ± 0.03	0.18 ± 0.02	.03	0.17 ± 0.04	0.16 ± 0.03
TpTe V_3_/QTc (ms)	0.18 ± 0.03	0.18 ± 0.02	.3	0.18 ± 0.04	0.19 ± 0.03
TpTe V_4_/QTc (ms)	0.17 ± 0.03	0.17 ± 0.02	.7	0.17 ± 0.04	0.17 ± 0.04
TpTe V_5_/QTc (ms)	0.17 ± 0.03	0.17 ± 0.02	.9	0.16 ± 0.03	0.17 ± 0.04
TpTe V_6_/QTc (ms)	0.16 ± 0.03	0.16 ± 0.02	.9	0.16 ± 0.03	0.17 ± 0.03
TpTe-dispersion/QTc-dispersion	0.71 ± 0.65	0.67 ± 0.43	.06	0.4 ± 0.28	0.77 ± 0.78

Values are presented as mean ± standard deviation.

MFS = Marfan syndrome; QTc = corrected QT; TpTe = T-peak–T-end; V_1_–V_6_ = electrocardiogram leads; VT = ventricular tachycardia.

### Outcomes in patients with MFS

During a median follow-up of 5.05 years (interquartile range 3.4–10.0 years), 9 patients with MFS (10%) died. None of the control patients died (Table 3) during a follow-up of 4.21 years (interquartile range 1.24–6.03 years) (Figure 1). Among patients with MFS, 2 (2%) died of heart failure, 3 (3%) of unknown causes, and (4%) of SCD. Of the 4 patients with SCD, 2 (50%) had already undergone aortic replacement surgery (David or Bentall procedure). Based on the documentation provided by the physician who pronounced death, there were no findings indicative of acute aortic dissection, which makes VT the more likely cause of death in patients with SCD.

**Table 3:** Clinical end points

Patient no.	End point events	Age at an end point event (y)	Sex	CVRF	Valvular disease or aortic surgery	LVEF	History of aortic dissection events in patients
1	Death (heart failure)	50	Male			1	
2	Death (heart failure)	78	Female	CAD, AHT		1	Type B dissection
3	Death (unknown)	69	Male		MVP	1	
4	Death (unknown)	32	Female		MVP, David	1	
5	Death (unknown), syncope	65	Male	AHT	MVR, Bentall	1	Type A dissection
6	SCD	70	Male	AHT		1	
7	SCD	26	Male		MVP	1	
8	SCD	47	Male	CAD	MVP, David	4	
9	SCD (VF), syncope, VT	42	Male		Bentall	4	Type A dissection
10	Syncope	41	Female	AHT	David	2	Type A dissection
11	Syncope	41	Male		Bentall	1	Type A + B dissection
12	Syncope	44	Female	AHT	MVP, David	1	
13	Syncope	34	Female		David	1	
14	Syncope	59	Female	AHT	MVP, MVR, David	1	
15	Syncope	59	Female		MVP, MVR	1	
16	Syncope	58	Female		David	1	
17	Syncope, VT	50	Female	AHT	MVP, Bentall	3	
18	VT	29	Male			1	

AHT = arterial hypertension; Bentall = Bentall procedure; CAD = coronary artery disease; CVRF = cardiovascular risk factors; David = David procedure; LVEF = left ventricular ejection fraction (1 = normal; 2 = mildly; 3 = moderate; 4 = severe); MVP = mitral valve prolapse; MVR = mitral valve repair; SCD = sudden cardiac death; VF = ventricular fibrillation; VT = ventricular tachycardia.

VT and syncope were observed in 3 (3%) and 10 (11%) patients with MFS, respectively, but not in the control group.

In total, 11 patients (12%) experienced the primary end point and 18 patients with MFS (20%) had at least 1 event including syncope. The clinical characteristics, underlying cardiac conditions, and procedures for patients who experienced an end point event are summarized in Table 3.

### Association of clinical parameters and ECG alterations with the combined end point of overall mortality or VT in patients with MFS

Patients with MFS and a combined primary end point (n = 11 [12.3%]) showed a longer QRS duration than did those with MFS without an end point (mean 119 ± 21 ms vs 103 ± 18 ms; *P* < .005). The ratio of TpTeD/QTD was 49% lower in patients with MFS and an end point (mean 0.42 ± 0.29 vs 0.81 ± 0.76; *P* = .038), and the mean ratio of TpTeD/QTcD was 0.4 ± 0.28 in patients with MFS and 0.76 ± 0.7 in control patients, respectively (*P* = .06).

ROC analysis revealed a cutoff value of 102 ms for QRS duration (sensitivity 90%; specificity 56%; AUC 0.77), 0.4 for the TpTeD/QTD ratio (sensitivity 63%; specificity 69%; AUC 0.69), and of 0.38 for the TpTeD/QTcD ratio (sensitivity 61%; specificity 69%; AUC 0.66).

Univariable Cox regression analysis identified several factors associated with the combined end point. These factors included the TpTeD/QTcD ratio (with a cutoff value of 0.39), male sex, left atrial (LA) dilatation, history of atrial fibrillation, and increased N-terminal pro–B-type brain natriuretic peptide levels. In contrast, homocysteine levels, QRS duration, and mitral valve prolapse (MVP) were not associated with this end point. Multivariable Cox regression analysis revealed N-terminal pro–B-type brain natriuretic peptide as the most influential parameter (hazard ratio 1.05 per 100-pg/mL increase) (Table 4). None of the other included characteristics, including the ECG parameters of ventricular dispersion, were associated with all-cause mortality or VT.

**Table 4:** Univariable and multivariable Cox regression analysis for the primary end point in patients with Marfan syndrome

Parameter	HR	95 % CI		Univariable *P*		Multivariable *P*
LA volume per 10 ml	1.35	1.12	1.64	.002	∗	.15
Male sex	5.01	1.05	23.87	.04	∗	.09
History of atrial fibrillation	9.51	2.51	36.01	.001	∗	.9
NT-proBNP level per 100 pg/ml	1.05	1.03	1.07	.00004	∗	.03
Homocysteine level per µmol/L	1.06	0.96	1.17	.250		
TpTe-QTc dispersion > 0.38	0.29	0.08	1.01	.05	∗	.8
Mitral valve prolapse	1.04	0.31	3.43	.950		
TpTe-QT dispersion > 0.4	0.34	0.10	1.18	.09		
QRS duration per 1 ms	1.02	0.98	1.05	.320		
TpTe maximum per 1 ms	0.9	0.94	1.05	.9		
TpTe V_5_ per 1 ms	1	0.9	1.07	.9		

The primary end point was all-cause mortality or ventricular tachycardia. ∗ = significant univariable parameter.

CI = confidence interval; HR = hazard ratio; LA = left atrial; NT-proBNP = N-terminal pro–B-type brain natriuretic peptide; QTc = corrected QT; TpTe = T-peak–T-end.

### Patients with MFS and syncope

During follow-up, syncope occurred in 10 patients with MFS (11%) with a mean age of 49 ± 10 years. The QT and PR intervals were prolonged (QT interval: 430 ± 30 ms vs 394 ± 34 ms; *P* = .007; PR interval: 200 ± 71 ms vs 161 ± 49 ms; *P* = .05). Hypertension (5 (50%) vs 8 patients (10%); *P* = .005) and history of atrial fibrillation (4 (40%) vs 8 patients (10%); *P* = .02) were more common in patients with MFS and syncope than in those without syncope. The mean LA volume was larger in patients with MFS and syncope than in those without syncope (86 ± 51 mL vs 51 ± 19 mL; *P* = .02). MVP was present in a total of 36 (40%) of patients in both groups. LVEF was normal in 7 (70%) and 73 (92%) of patients with MFS with and without syncope (*P* = .06).

In patients with MFS and syncope, the TpTe interval was prolonged (80 ± 11 ms vs 71 ± 10 ms; *P* = .05) and the TpTe/QT ratio shortened (0.16 ± 0.03 vs 0.19± 0.03; *P* = .006). ROC analysis for TpTe and TpTe/QT ratio revealed cutoff values of 82 ms (sensitivity 62.5%; specificity 85%; AUC 0.73) and 0.17 (sensitivity 70%; specificity 81%; AUC 0.78), respectively.

## Discussion

### Main findings

Our study presents an exploratory evaluation of the clinical, echocardiographic, and ECG characteristics of patients with MFS compared to a control group. The results reveal several differences between the 2 groups, particularly in terms of ECG parameters and clinical outcomes. Specifically, patients with MFS exhibited longer QRS duration, repolarization, and TpTe times than did the control group. The incidence of SCD was higher in the studied population, and affected patients were younger, as expected when compared to prevalence studies in the general population. In addition, patients with a combined end point of all-cause mortality or VT exhibited disturbances in intraventricular propagation as well as altered dispersion and repolarization. In patients with MFS and syncope, we observed significant changes in cardiac structure, cardiovascular risk factors, and irregularities in repolarization.

### Patients with MFS have higher rates of mortality

During a mean follow-up of 5.5 years, the mortality rate was 10%. This is higher than expected compared to the general population.^14^ Several factors contribute to the increased mortality rate in patients with MFS, including aortic dissection, ventricular arrhythmias, and heart failure.^15^^,^^16^

Our study observed a higher incidence of VT, syncope, and SCD in this cohort of patients with MFS, indicating an increased risk of life-threatening arrhythmias. The rate of SCD, which was 4%, was similar to that reported in previous studies.^3^

The control group, which consisted mainly of young and healthy individuals, did not receive routine rhythm monitoring comparable to that of the MFS group. This difference in monitoring approach may have led to underdetection of arrhythmias or end point events in the control population. Nevertheless, compared to studies in the general population, the higher frequency of rhythm and end point events in the MFS group is clinically relevant.

### Dispersion and repolarization in patients with MFS

In this study, patients with MFS showed significantly longer QRS duration, QT interval, QTc interval, and TpTe interval than did controls. These longer intervals may indicate altered depolarization and repolarization, as well as increased dispersion, potentially raising the risk of ventricular arrhythmias. This, in turn, may contribute to the higher incidence of events such as SCD and VTs.^8^

Data on the impact of body habitus on cardiac depolarization and repolarization are scarce. One study reported that patients with MFS tend to have more vertical QRS axis and noted pathological T-wave inversions in approximately one-fifth of the cohort.^17^ Although increased body mass index has been associated with prolonged QT interval, TpTe interval, and QRS duration, our cohort of patients with MFS had a normal body mass index (Table 1), suggesting that body composition is an unlikely confounder.^18^ Tikkanen et al^19^ recently conducted a study that linked QRS prolongation to SCD in a cohort of more than 20,000 patients in Finland. However, they did not find any association with the QT interval. We found that patients with MFS who experienced an end point event had a longer QRS duration. However, Cox regression analysis did not show any association with the composite end point of all-cause mortality or VT, which could be due to reduced statistical power revealed by the post hoc power analysis for this parameter.

The link between TpTe and SCD is still a matter of debate. TpTe has been identified as a marker of arrhythmogenic events in various patient populations, including those with hypertrophic cardiomyopathy, Brugada syndrome, or reduced LVEF due to any cause.^20^, ^21^, ^22^ Yamaguchi et al^13^ found that the TpTe/QT ratio can predict torsades de pointes tachycardias in patients with long QT syndrome. However, Michalek et al^23^ studied 243 patients with implantable cardioverter-defibrillators and reduced LVEF after myocardial infarction and found no association between survival and TpTe or corrected TpTe intervals. The TpTe and TpTe/QT ratio did not predict SCD in patients with end-stage renal disease.^9^ Finally, Porthan et al^24^ found no association between TpTe and SCD in the general population.

Watanabe et al^25^ used body surface ECG to analyze intraventricular repolarization and dispersion in their study. However, they did not find a correlation between the TpTe interval and transmural dispersion of repolarization. Kors et al^5^ used TpTeD as a measure of vectorcardiogram T-loop deformity in an in silicio model. However, they also noted that pathological T-loops can correspond to normal TpTe or TpTeD values, limiting their sensitivity.

In our cohort, TpTe alone did not predict worse outcomes. However, a low ratio of TpTeD/QTcD was associated with worse outcomes in univariable analysis. In addition, TpTe was prolonged in patients with MFS and syncope. These findings suggest that these altered ECG parameters, reflecting repolarization and dispersion, are associated with severely affected patients with MFS.

### Association of structural alterations of the heart with arrhythmogenic events in patients with MFS

Our study confirms that patients with MFS have larger left ventricular end-diastolic diameter and LA volume than do controls. These findings are consistent with previous literature and are likely because of the connective tissue abnormalities associated with MFS, which can cause aortic root dilatation, MVP, and atrial enlargement.^26^^,^^27^

Taub et al^28^ reported MVP in 28% of a cohort of 90 patients with MFS. Demolder et al^29^ conducted a retrospective analysis and found a 34% prevalence of MVP in their cohort along with an association with mitral annular disjunction and VT. In our cohort of patients with MFS, the prevalence of MVP was approximately 40%. However, we did not find any association between MVP and VT or SCD. Since this was a retrospective study, we did not have specific data on the prevalence of mitral annular disjunction. As there were many syncope events in the cohort of patients with MFS (n = 10 [11%]), including 1 patient with VT and 2 deaths during follow-up, clinical implications include being vigilant about syncope events and considering implantable loop recorder implantation in patients with MFS, especially those with a history of syncope.

### Limitations

Our study has several limitations. First, this was a retrospective study conducted at a single center. Second, although relatively large for a cohort of patients with MFS, the limited sample size results in reduced statistical power, which restricts the use of meaningful multivariable analyses. Furthermore, as no postmortem autopsies were performed, the cause of death was determined on the basis of clinical data, which may have led to misclassification.

## Conclusion

Our study shows that ECG parameters of repolarization and dispersion are altered in patients with MFS, but their role in risk stratification may be limited.
